# Antibacterial Activity of Selected Essential Oil Compounds Alone and in Combination with β-Lactam Antibiotics Against MRSA Strains

**DOI:** 10.3390/ijms21197106

**Published:** 2020-09-26

**Authors:** Paweł Kwiatkowski, Łukasz Łopusiewicz, Agata Pruss, Mateusz Kostek, Monika Sienkiewicz, Radosław Bonikowski, Iwona Wojciechowska-Koszko, Barbara Dołęgowska

**Affiliations:** 1 Department of Diagnostic Immunology, Chair of Microbiology, Immunology and Laboratory Medicine, Pomeranian Medical University in Szczecin, Powstańców Wielkopolskich 72, 70-111 Szczecin, Poland; iwonakoszko@interia.pl; 2 Center of Bioimmobilisation and Innovative Packaging Materials, Faculty of Food Sciences and Fisheries, West Pomeranian University of Technology Szczecin, Janickiego 35, 71-270 Szczecin, Poland; lukasz.lopusiewicz@zut.edu.pl (Ł.Ł.); mkosa9406@gmail.com (M.K.); 3 Department of Laboratory Medicine, Chair of Microbiology, Immunology and Laboratory Medicine, Pomeranian Medical University in Szczecin, Powstańców Wielkopolskich 72, 70-111 Szczecin, Poland; agata.pruss@pum.edu.pl (A.P.); barbara.dolegowska@pum.edu.pl (B.D.); 4 Department of Allergology and Respiratory Rehabilitation, Medical University of Łódź, Żeligowskiego 7/9, 90-752 Łódź, Poland; monika.sienkiewicz@umed.lodz.pl; 5 Institute of General Food Chemistry, Faculty of Biotechnology and Food Sciences, Łódź University of Technology, Stefanowskiego 4/10, 90-924 Łódź, Poland; radoslaw.bonikowski@p.lodz.pl

**Keywords:** MRSA, essential oil compounds, β-lactam antibiotics, checkerboard assay, FTIR

## Abstract

This study aimed to determine the effect of selected essential oil compounds (EOCs) on the antibacterial activity of β-lactam antibiotics (βLAs) against methicillin-resistant *Staphylococcus aureus* (MRSA) strains. The following parameters were studied: antibiotic susceptibility testing, detection of *mecA* gene and evaluation of genotypic relativity of isolates using molecular techniques, analysis of chemical composition applying Fourier-transform infrared (FTIR) spectroscopy, and determination of antibacterial activity of EOCs alone and in combination with βLAs against MRSA strains using microdilution and checkerboard methods. It was found that all isolates expressed MRSA and resistance phenotypes for macrolides, lincosamides, and streptogramins B. All isolates harbored the *mecA* gene and belonged to three distinct genotypes. Eight of the 10 EOCs showed efficient antimicrobial activity against the MRSA reference strain. The analysis of interaction between EOCs and βLAs against the MRSA reference strain revealed a synergistic and additive effect of the following combinations: methicillin (Met)-linalyl acetate (LinAc), penicillin G (Pen)-1,8-cineole (Cin), and Pen-LinAc. Analysis of EOC-βLA interactions showed a synergistic and additive effect in the following combinations: Met-LinAc (against low- and high-level βLAs resistance strains), Pen-Cin, and Pen-LinAc (against low-level βLAs resistance strains). It was also confirmed that changes in phosphodiester, -OH, -CH_2_ and -CH_3_ groups may change the interactions with βLAs. Moreover, the presence of two CH_3_O- moieties in the Met molecule could also play a key role in the synergistic and additive mechanism of LinAc action with Met against MRSA strains. Direct therapy using a Met-LinAc combination may become an alternative treatment method for staphylococcal infections caused by MRSA. However, this unconventional therapy must be preceded by numerous cytotoxicity tests.

## 1. Introduction

*Staphylococcus aureus* is the most important and best-known species from the *Staphylococcus* genus. It asymptomatically colonizes the skin and mucous membranes, while it is also the cause of many infections occurring in the hospital environment [1]. It is notorious due to its ability to become resistant to many antibiotics. Some examples that could be quoted are resistance to penicillin—associated with the production of penicillinase (an enzyme hydrolyzing the β-lactam ring), and methicillin—associated with the production of an altered penicillin-binding protein (PBP) 2A, PBP2A (which has a reduced affinity toward β-lactam antibiotics) [2,3]. The *mecA* gene, which is part of the staphylococcal chromosomal cassette *mec* (SCC*mec*) [3], is responsible for PBP2A production. At present, there are 13 (I–XIII) types of SCC*mec* [4]. It is noteworthy that methicillin-resistant *S. aureus* (MRSA) strains are not only associated with the hospital environment (HA-MRSA, hospital-acquired methicillin-resistant *S. aureus*), but can also occur in outpatients (CA-MRSA, community-acquired methicillin-resistant *S. aureus*). CA-MRSA infections mostly affect the skin and soft tissues. These strains are characterized by greater virulence (e.g., they have Panton–Valentine leukocidin), but also tend to be sensitive to most antibiotics. The HA-MRSA strains are most often isolated from long-term hospitalized patients with chronic diseases. HA-MRSA shows resistance to several groups of antibiotics (macrolides, clindamycin, quinolones, tetracyclines, aminoglycosides, and trimethoprim/sulfamethoxazole). The widespread use of antibiotics leads to the emergence of resistance [5], whereby the spread of MRSA is a global problem present on all continents. Monotherapy is becoming noneffective and the new treatment options rely on combining selected antibiotics, e.g., daptomycin + ceftaroline. Preventive actions against MRSA are widely implemented across healthcare facilities and, among other goals, they are aimed at limiting the emergence of MRSA via prudent use of antimicrobial agents, preventing MRSA transmission between patients, and preventing the development of infection in carriers by decolonization. According to literature data, because of increasing mupirocin resistance, other strategies are recommended such as vaccines, bacteriophages, or bacteriophage-derived lytic proteins [5,6]. On the other hand, resistance to biocides is also becoming more common, such as chlorhexidine and octenidine dihydrochloride [7]. Generally speaking, it is a fact that searching for new alternative approaches tackling resistance to antibiotics or biocides is reasonable and necessary.

Secondary metabolites of plants like alkaloids, terpenes, flavonoids, quinones, or resins, obtained from many plant species, possess unique activities that are used in many branches of industries such as cosmetic or food, making them interesting examples. Many of them show antibacterial activity against pathogens, including multidrug-resistant bacteria (e.g., MRSA or phenotypes expressing resistance to macrolides, lincosamides, and streptogramins B (MLS_B_)) [8]. It is known that terpenes constitute the largest group of natural compounds. Terpenic raw materials include essential oils (EOs) and are made up mostly of monoterpenes and sesquiterpenes. Some derivatives of monoterpenes are chemical compounds such as alcohols, esters, phenols, ketones, and aldehydes [9]. According to the literature data, monoterpene derivatives from plants have antistaphylococcal potential [10,11]. Moreover, it is known that the combination of secondary metabolites with antimicrobial agents can show synergistic or additive effects; thus, it is possible to use these combinations to reduce the effective dose of chemical antimicrobials and antiseptic agents [12,13].

Hence, the aim of the present study was to investigate the effect of selected essential oil compounds (EOCs: 1,8-cineole, eugenol, carvacrol, linalool, linalyl acetate, *trans*-anethole, thymol, menthone, menthol, β-caryophyllene) on the antibacterial activity of β-lactam antibiotics (methicillin, penicillin G) against MRSA strains. Special attention was paid to the following parameters: (i) optimization—evaluating the best combination (showing synergistic and additive effects) of the analyzed EOCs with β-lactam antibiotics against the MRSA reference strain using the checkerboard method, and (ii) evaluating the effectiveness of combinations of selected EOCs with β-lactam antibiotics against MRSA isolates using the checkerboard method. Furthermore, the study also implemented antibiotic susceptibility testing, detection of *mecA* gene using the polymerase chain reaction (PCR) method, molecular typing using the pulsed-field gel electrophoresis (PFGE) method, and analysis of the chemical moieties in *S. aureus* cells using Fourier-transform infrared (FTIR) spectroscopy.

## 2. Results

### 2.1. Antimicrobial Susceptibility Testing (AST) Results

Characteristics of *S. aureus* strains are summarized in Table 1. It was confirmed that *S. aureus* ATCC 43300 was susceptible to ciprofloxacin (CIP), but resistant to gentamicin (GE), cefoxitin (FOX), erythromycin (E), and clindamycin (CC) (MRSA and phenotypes expressing resistance to constitutive macrolides, lincosamides, and streptogramins B (cMLS_B_)). Nevertheless, all isolates were resistant to GE, CIP, and FOX. Moreover, the D-test confirmed the following resistance phenotypes in the isolates: cMLS_B_ (87.5%, *n* = 7) and inducible MLS_B_ (iMLS_B_) (12.5%, *n* = 1).

### 2.2. Presence of MecA Gene and PFGE Results

**Figure 1:**
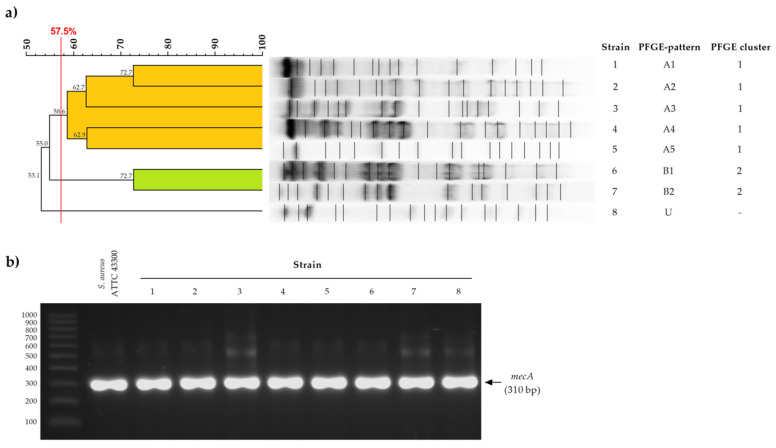
Pulsed-field gel electrophoresis (**a**) and polymerase chain reaction amplification of *mecA* gene; (**b**) results of methicillin-resistant *Staphylococcus aureus* strains. U—unique strain.

Molecular analysis showed three distinct PFGE macrorestriction banding patterns (A, B, and unique—U) and two clusters (1 and 2) among the eight MRSA isolates (Figure 1a).

Both MRSA reference strains and all isolates harbored the *mecA* gene encoding penicillin-binding protein 2A (PBP2A) (Figure 1b).

### 2.3. Optimization Assay Results

The optimization assay results showed that the *S. aureus* ATCC 43300 strain was sensitive to almost all EOCs, excluding menthol (>445.0 mg/mL) and β-caryophyllene (>450.5 mg/mL). The highest inhibitory activity against the reference strain was observed for thymol. It was also confirmed that the reference strain was resistant to both methicillin and penicillin G. Results of minimal inhibitory concentrations (MICs) of both EOCs and β-lactam antibiotics against the reference strain are listed in Table 2.

The study indicated that linalyl acetate showed a synergistic and an additive effect with methicillin (fractional inhibitory concentration index, FICI = 0.4) and penicillin G (FICI = 0.6), respectively. It was also noted that the combination of 1,8-cineole and penicillin G (FICI = 0.1) revealed synergism. Overall, a number of combinations showed an indifferent effect, excluding the following combinations: methicillin + thymol (FICI = 5.0, antagonism) and methicillin + menthone (FICI = 6.0, antagonism). Due to the lack of MIC results for menthol and β-caryophyllene, the checkerboard assay using these compounds was omitted. Moreover, it was also found that Mueller–Hinton broth (MHB) containing 1% (*v*/*v*) Tween 80 or 2% (*v*/*v*) dimethyl sulfoxide (DMSO) had no impact on the growth of the reference strain. Results for the checkerboard assay (FICI values) against *S. aureus* ATCC 43300 reference strain are presented in Table 2.

According to the optimization assay, only the following combinations were studied on all MRSA isolates: methicillin + linalyl acetate, penicillin G + 1,8-cineole, and penicillin G + linalyl acetate (showing a synergistic or additive effect against the reference strain).

### 2.4. Antibacterial Effects of Selected Combinations of EOCs and β-Lactam Antibiotics against MRSA Isolates

The results confirmed that all MRSA isolates were resistant to both methicillin and penicillin G. The MIC of β-lactam antibiotics ranged from 7.8 ± 0.0 mg/L to 1000.0 ± 0.0 mg/L and from 3.9 ± 0.0 mg/L to 125.0 ± 0.0 mg/L, respectively, for methicillin and penicillin G. In turn, the MICs of 1,8-cineole and linalyl acetate against all isolates ranged from 28.8 ± 0.0 mg/mL to 57.6 ± 0.0 mg/mL and from 28.2 ± 0.0 mg/mL to 112.6 ± 0.0 mg/mL, respectively. Results of the MIC values of β-lactam antibiotics and EOCs against MRSA isolates are summarized in Table 3.

The study indicated that linalyl acetate showed synergistic (*n* = 2; FICI = 0.2–0.4) and additive (*n* = 6; FICI = 0.6–1.0) activities with methicillin against MRSA isolates. A decrease in methicillin MIC values from 1000 mg/L to 62.5 mg/L was noted. It was also proven that strains with an MIC value for penicillin G = 3.9 ± 0.0 mg/L showed synergistic and additive effects in combination with 1,8-cineole (FICI = 0.2–0.4) and linalyl acetate (FICI = 0.6–0.7), respectively. In contrast, indifference effect was found for all strains that exhibited an MIC value for penicillin G ≥31.3 ± 0.0 mg/L. Results for the checkerboard assay (FICI values) against MRSA isolates are listed in Table 3.

### 2.5. FTIR Analysis

**Figure 2:**
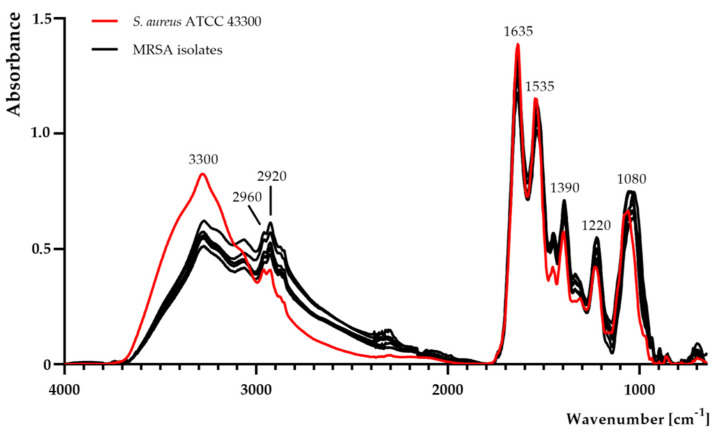
Fourier-transform infrared spectroscopy analysis of *Staphylococcus aureus* ATCC 43300 and methicillin-resistant *S. aureus* (MRSA) isolates.

Complete FTIR spectra of the samples are shown in Figure 2. The qualitative and quantitative differences between the MRSA reference strain and MRSA isolates were observed. A noticeable decrease in absorbance at band 3300 cm^−1^ was detected in MRSA isolates. Moreover, the absorbance of MRSA isolates increased at 2960 cm^−1^, 2920 cm^−1^, and 1390 cm^−1^.

**Figure 3:**
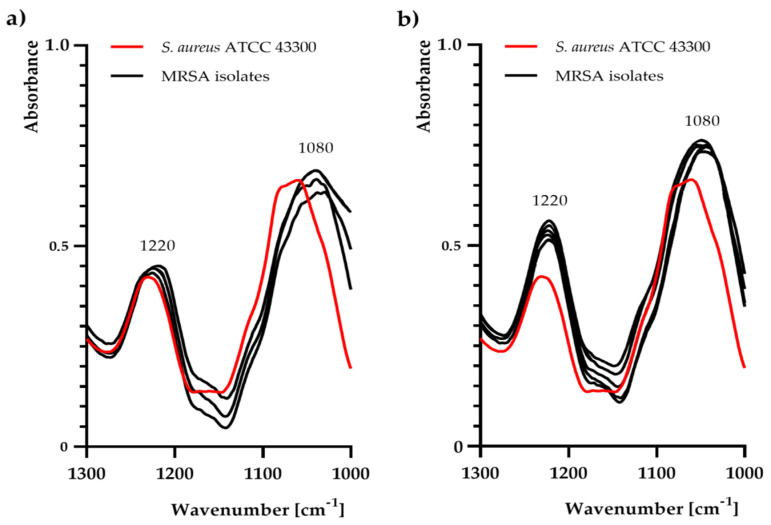
Fourier-transform infrared spectroscopy analysis (in the range of 1300–1000 cm^−1^) for *Staphylococcus aureus* ATCC 43300 and methicillin-resistant *S. aureus* (MRSA) isolates: (**a**) No. 4, 6, and 8; (**b**) No. 1, 2, 3, 5, and 7.

In particular, noticeable changes were observed at 1220 cm^−1^ and 1080 cm^−1^ (Figure 2, Figure 3). In contrast to the control sample (MRSA reference strain), there was a slight increase in absorbance at 1220 cm^−1^ and 1080 cm^−1^ only in the following isolates: No. 1, 2, 3, 5, and 7.

## 3. Discussion

Currently, selecting the most appropriate treatment method for patients suffering from infections caused by multidrug-resistant *S. aureus* strains is a worldwide growing problem. Drugs used in hospital antibiotic therapy (e.g., β-lactam antibiotics) against MRSA strains are excluded from treatment, whereas the use of other antibiotics should be monitored due to their undesirable side effects (e.g., ototoxicity, nephrotoxicity, or hematological disorders caused by vancomycin). Moreover, due to the ineffectiveness of newly synthesized antibiotics, as well as the negative resulting modification of “old” drugs, therapeutic options are very narrow. Admittedly, various attempts are being made to control the multidrug resistance of bacterial strains, such as a combination of antibiotic therapy [14], short therapy using high doses of antibiotics, shortening hospital stays, and even screening for alarm pathogens [15], which are unfortunately without a spectacular effect. These activities are primarily based on enforcing the compliance with the principles of rational antibiotic therapy in hospitals.

According to the literature data, studies on the interaction between antibiotics and other compounds (e.g., natural product-derived) could represent “a new era of phytopharmaceuticals” and a promising strategy in the treatment and control of infection caused by multidrug-resistant bacteria [16]. Essential oils (EOs) and essential oil compounds (EOCs) possessing antimicrobial activity (mainly via their action through the disruption of the bacterial cell membrane), showing synergistic or additive effects with antibiotics or biocides, are the best examples of the power of natural substances. There have been reports of attempts to synergistically enhance the action of antibacterial drugs under the pressure of EOs and EOCs. Boonyanugomol et al. [17] reported that EO from *Zingiber cassumunar* RoxB exhibited a synergistic effect with aminoglycosides (gentamicin, amikacin), tetracyclines (doxycyline, tetracycline), and fluoroquinolones (ciprofloxacin, levofloxacin) against extensively drug-resistant *Acinetobacter baumannii*. Mahboubi and Bidgoli [18] found a synergistic effect of *Zataria multiflora* EO in combination with vancomycin against *S. aureus* strains isolated from infection sites. On the other hand, our previous studies also indicated that peppermint and caraway EOs revealed synergistic activity against gentamicin-intermediate (producing extended-spectrum β-lactamase (ESBL) and New Delhi metallo-β-lactamase-1 (NDM-1)) and gentamicin-resistant (producing ESBL) *Klebsiella pneumoniae* strains, respectively [19]. Furthermore, Uzair et al. [20] demonstrated that *Origanum vulgare* and *Mentha pulgeium* EOs showed effective synergistic activity in combination with amoxicillin against MRSA strains. It was also proven that carvacrol and cuminaldehyde synergistically enhanced the antibacterial activity of vancomycin against vancomycin-susceptible *Enterococcus faecium* and vancomycin-resistant *E. faecium* isolates [21]. The study performed by Aelenei et al. [22] revealed synergistic interactions of coriander EO with amoxicillin and gentamicin against MRSA reference strains (ATCC 43300, ATCC 33591). These authors also proved that linalool demonstrated synergistic interactions with amoxicillin, oxacillin, gentamicin, ciprofloxacin, and tetracycline against these reference strains. Interestingly, in the present study, linalool exhibited indifferent effects with both methicillin and penicillin G against *S. aureus* ATCC 43300. These effects could be related to the different molecular structures of these β-lactam antibiotics and their direct interaction with staphylococcal cells.

The use of EOCs in combination with antibiotics for the treatment of infection has also been gaining popularity. Taking this into account, we decided to investigate the potential for a synergistic or additive effect of the selected EOCs with β-lactam antibiotics. Special attention was paid to the following combinations against MRSA isolates: methicillin + linalyl acetate, penicillin G + 1,8-cineole, and penicillin G + linalyl acetate (obtained after an optimization assay using the MRSA reference strain). 1,8-Cineole (also known as eucalyptol) is a safe monoterpene present in many EOs such as *Eucalyptus globulus* EO, which is recommended in the alternative therapy of respiratory tract infections [23,24]. Our results showed that combination of 1,8-cineole and penicillin G exhibited a synergistic effect only against MRSA strains with an MIC of penicillin G = 3.9 ± 0.0 mg/L. Similar results were reported by other authors. Remmal and Akhmouch [25] patented a pharmaceutical formulation of 1,8-cineole with amoxicillin showing synergism. Moreover, Hriouech et al. [26] proved the synergistic activity of β-lactam antibiotics (amoxicillin/clavulanic acid, AMC) in combination with 1,8-cineole against an MRSA strain. Interestingly, the MIC for AMC against the MRSA strain was 1 mg/L, but these authors did not focus on MRSA strains with high-level β-lactam antibiotic resistance. Linalyl acetate is a safe monoterpene present in many EOs, such as lemon, clary sage, or lavender (LEO). According to the literature, there are scarce data on the interaction between linalyl acetate and antibiotics against *S. aureus* strains. For instance, our previous study showed the indifferent effect of linalyl acetate in combination with mupirocin against mupirocin-susceptible and laboratory-induced low-level mupirocin-resistant MRSA strains [11]. Nevertheless, LEO is recommended for the treatment of many skin infections caused by *S. aureus* such as carbuncles, abscesses, and wounds [27]. Moreover, our other previous studies showed synergism between LEO and octenidine dihydrochloride against MRSA strains [13]. Taking that fact into account, linalyl acetate was chosen for the current study. Obtained results showed synergistic and additive activities in combination with methicillin and penicillin G against the MRSA reference strain, respectively. It was also proven that linalyl acetate exhibited a synergistic or additive effect in combination with methicillin against all MRSA isolates (both low- and high-level β-lactam antibiotic-resistant strains). Interestingly, an additive effect of the penicillin G-linalyl acetate combination, against only three of eight MRSA isolates with an MIC of penicillin G = 3.9 ± 0.0 mg/L, was observed. Moreover, these three MRSA isolates belonged to three different PFGE patterns.

On the basis of the results obtained, it was found that MRSA strains (reference strain and isolates) with an MIC of penicillin G = 3.9 ± 0.0 mg/L showed the synergistic or additive effects of both 1,8-cineole and linalyl acetate with this β-lactam antibiotic. However, MRSA isolates with an MIC of penicillin G ≥31.3 ± 0.0 mg/L (also possessing high-level resistance to methicillin—1000 mg/L) exhibited an indifferent or antagonistic effect with penicillin G. Similar results were reported in our previous study [19]. It was proven that *K. pneumoniae* strains with an MIC of gentamicin ≤3.1 ± 0.0 mg/L and >150 mg/L exhibited a synergistic and indifferent effect with peppermint EO, respectively. According to Trombetta et al. [28], monoterpenes (such as linalyl acetate, thymol, or (+)-menthol) can cause a perturbation of the lipid fraction of the bacterial cell membrane. Lopez-Romero et al. [29] came to the same conclusion in their study of the antimicrobial mode of action of EOCs (carveol, carvone, citronellol, and citronellal) against *Escherichia coli* and *S. aureus*. They noted an action of EOCs on the cell surface, as well as the bacterial membrane.

In the current study, deep analysis of particular functional groups in whole MRSA cells using FTIR spectroscopy was performed. Detection and identification of microorganisms using spectroscopic techniques is a promising and inexpensive approach due to its sensitivity, simplicity, and time- and cost-effectiveness. Spectroscopic tools not only provide competitive and rapid identification methods, but also allow investigating microorganisms in their intact state. These also appear to be very promising tools to study microbial metabolism, antibiotic susceptibility, and other interactions with drugs [11,13,30]. Special attention was paid to the analysis of bands correlated with structures present in the MRSA cell wall (e.g., peptidoglycan layer). Regarding FTIR analysis, a noticeably higher band at 3300 cm^−1^, suggesting more -OH groups in the MRSA reference strain, was observed. Moreover, a noticeable growth in absorbance in the region 2920–2960 cm^−1^, attributed to -CH_2_ and -CH_3_ groups in MRSA isolates was recorded [11]. Interestingly, at 1220 cm^−1^ and 1080 cm^−1^, the higher intensity in MRSA isolates (with high-level methicillin (MIC = 1000 ± 0.0 mg/L) and penicillin G (MIC ≥ 31.3 ± 0.0 mg/L) resistance strains) can be attributed to the symmetric and asymmetric PO_2_^−^ stretching vibrations in phosphodiester groups, present, among others, in teichoic acid/lipoteichoic acid of Gram-positive bacteria [31]. The above-mentioned changes may affect the electrostatic interactions with antibacterial molecules, as also proven in our previous studies [11,13]. Moreover, blaR1 protein (which is involved in sensing of the β-lactam antibiotics and then transduction of the information to the cell’s interior) may play an important role in the association with -OH groups [32]. Hence, changes in the phosphodiester, as well as the -OH, -CH_2_, and -CH_3_, groups may lead to a change in the electrostatic potential of the cell wall, thereby hindering attachment of antibiotics to protein receptors. On the other hand, the presence of two methoxy (CH_3_O-) moieties in the methicillin molecule could also play a key role in the synergistic and additive mechanism of action of linalyl acetate with methicillin against MRSA strains. Nevertheless, further analysis is still required to understand this complex mechanism of action.

## 4. Materials and Methods

### 4.1. Bacterial Strains and Growth Condition

Eight *S. aureus* strains belonging to the Chair of Microbiology, Immunology, and Laboratory Medicine, Pomeranian Medical University in Szczecin (Poland) collection were analyzed in the study. The strains were isolated from different inpatients from the following sources: surgical wound (*n* = 5), bronchoalveolar lavage (BAL; *n* = 2), and urine (*n* = 1). The bacteria were cultured on Columbia agar with 5% sheep blood (bioMérieux, Warsaw, Poland) and incubated for 18 h at 37 °C in aerobic atmosphere. All isolates were identified using basic assays (positive catalase and coagulase tests, as well as a biochemical test with the use of GP-card on VITEK 2 Compact (bioMérieux, Warsaw, Poland)), which confirmed their affiliation with the *S. aureus* species.

*S. aureus* ATCC 43300 was used as a control for *mecA* gene and in the optimization assay to obtain the best combination of essential oil compound (EOC) with β-lactam antibiotic.

### 4.2. Chemicals

**Figure 4:**
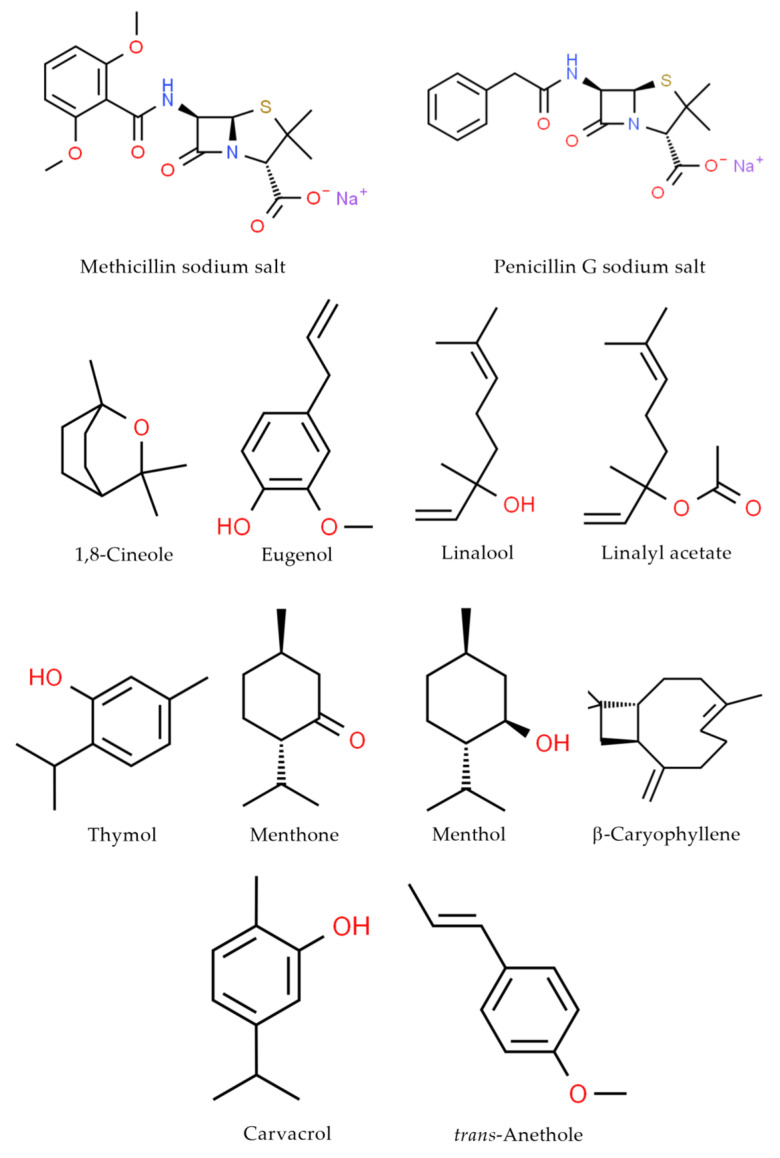
Chemical structures of the compounds used in this study.

The β-lactam antibiotics (Figure 4) methicillin sodium salt (≥95% purity) and penicillin G sodium salt (>96% purity) were purchased from Sigma-Aldrich (Darmstadt, Germany). Concentrations of the antibiotics from 1000 to 0.12 mg/L were prepared by dissolving the medicines in 2% (*v*/*v*) dimethyl sulfoxide (DMSO, Loba Chemie, Mumbai, India) and diluting with Mueller–Hinton broth (MHB; Sigma-Aldrich, Darmstadt, Germany).

The ten EOCs (Figure 4) used in this study, 1,8-cineole (99% purity), eugenol (≥98% purity), linalool (≥96% purity), linalyl acetate (≥97% purity), thymol (≥99% purity), menthone (98% purity), menthol (>99% purity), and β-caryophyllene (>95% purity), were purchased from ErnestoVentós, S.A. (Barcelona, Spain), whereas carvacrol (99% purity) and *trans*-anethole (99% purity) were obtained from Sigma-Aldrich (Darmstadt, Germany). Concentrations of the EOCs from 500 to 0.12 µL/mL were prepared by dissolving constituents in 1% (*v*/*v*) Tween 80 (Sigma-Aldrich, Darmstadt, Germany) and diluting with MHB. Using the known densities of EOCs, the results were expressed in mg/mL.

### 4.3. Antibiotic Susceptibility Testing (AST) Assay

The AST assay of *S. aureus* isolates with respect to gentamicin (GE), ciprofloxacin (CIP), and cefoxitin (FOX, determination of MRSA phenotype) was estimated using the Kirby–Bauer method, whereas the erythromycin (E) and clindamycin (CC) (determination of phenotypes expressing resistance to macrolides, lincosamides, and streptogramins B (MLS_B_)) assays were estimated using the D-test. All tests were performed according to European Committee on Antimicrobial Susceptibility Testing (ECUAST) recommendations [33]. The AST assay was conducted on Mueller–Hinton agar (bioMérieux, Warsaw, Poland) cultured with *S. aureus* (0.5 McFarland scale). Then, the antibiotic discs (Diag-Med, Warsaw, Poland) were placed on MHA apart (for GE, CIP, FOX) and approximately 12–20 mm apart (edge to edge) (for E, CC). After 18 h of incubation at 37 °C, the results were interpreted according to the EUCAST recommendations [33]. The results of the D-test were evaluated for each *S. aureus* strain and expressed as constitutive MLS_B_ (cMLS_B_), inducible MLS_B_ (iMLS_B_—the D phenomenon), or MS_B_ resistance phenotype.

### 4.4. MecA Gene Detection

#### 4.4.1. DNA Isolation

All MRSA isolates were cultured on Columbia agar with 5% sheep blood and incubated for 18 h at 37 °C. After incubation, one colony of each strain was transferred to an Eppendorf tube containing 1 mL of tryptic soy broth (Sigma-Aldrich, Darmstadt, Germany) and re-incubated for 18 h at 37 °C. Next, Eppendorf tubes were centrifuged for 5 min at 300× *g*, and the obtained pellet was washed twice using phosphate-buffered saline (PBS, pH 7.4). Finally, genomic DNA was isolated from the pellet using the GeneMatrix Bacterial and Yeast Genomic DNA Purification Kit (EURx, Gdansk, Poland), according to the manufacturer’s protocol.

#### 4.4.2. PCR Amplification

PCR amplification of *mecA* gene was performed with a pair of specific primers: mecA1 (5′–GTA GAA ATG ACT GAA CGT CCG ATA A–3′) and mecA2 (5′–CCA ATT CCA CAT TGT TTC GGT CTA A–3′) [34]. PCR was performed using a StartWarm HS-PCR Mix mixture (A&A Biotechnology, Gdynia, Poland) as described in our previous study [35]. Amplification was conducted using an Applied Biosystems Veriti 96 Well Thermal Cycler (Applied Biosystems, Norwalk, CT, USA) with the following protocol: 95 °C for 4 min, then 35 cycles of 95 °C for 15 s, 53 °C for 30 s, and 72 °C for 60 s, followed by 72 °C for 7 min. PCR assay runs incorporated a reagent control with *S. aureus* ATCC 43300 (*mecA*-positive). The PCR products were analyzed by electrophoresis (100 V, 60 min, 1× Tris/borate/ethylenediaminetetraacetic acid—TBE) in 1.5% agarose gel (DNA Gdansk, Gdansk, Poland) containing 0.5 μg/mL of ethidium bromide (Sigma-Aldrich, Darmstadt, Germany), then visualized and photographed using a gel image system (GelDoc-It2 Imager, Analityk Jena US LLC, Upland, CA, USA).

### 4.5. Macro-Restriction Analysis of Genomic DNA of MRSA Isolates

All MRSA isolates were characterized using PFGE. Preparation of bacterial DNA was conducted on the basis of the GenePath Group 6 Reagent Kit Instruction Manual (Bio-Rad, Marnes-la-Coquette, France) using CHEF Bacterial Genomic DNA Plug Kits (Bio-Rad, Marnes-la-Coquette, France). Digestion of DNA was performed using *Sma*I (ThermoScientific, Waltham, MA, USA) according to the protocol described by the manufacturer. PFGE was carried out with a CHEF DR III apparatus (Bio-Rad, Marnes-la-Coquette, France) in 1.2% agarose gel (DNA Gdansk, Gdansk, Poland) and 1× TBE buffer with the following parameters: 6 V/cm at 14 °C, initial switching time—2.2 s, final switching time—54.2 s, including angle—120°, run time—22 h. The *Sma*I digesting DNA from *S. aureus* ATCC 43300 was included as a normalization standard. After electrophoresis, the gel was stained with ethidium bromide (0.5 μg/mL), and then visualized and photographed applying a gel image system (GelDoc-It2 Imager, Analityk Jena US LLC, USA).

The obtained restriction PFGE profiles were analyzed using the FPQuest program (Bio-Rad, Marnes-la-Coquette, France). Classification of individual restriction patterns for particular genetic profiles was carried out using the unweighted pair group method with arithmetic mean (UPGMA) (similarity coefficient (S_AB_) = 57.5%) and the Dice coefficient (2.0%). The PFGE results are shown in the form of a dendrogram.

### 4.6. Combination of EOCs with β-Lactam Antibiotics—Optimization Assay

To obtain the best combination of EOCs with antibiotics, an optimization assay using *S. aureus* 43300 was performed. The first step of the optimization assay consisted of determination of minimal inhibitory concentration (MIC) of chemicals using the broth microdilution method according to the Clinical and Laboratory Standards Institute, with slight modification as described elsewhere [11]. Briefly, each well contained the appropriate concentration of EOC (50 μL) or β-lactam antibiotic (50 μL) and 50 μL of staphylococcal suspension (10^6^ CFU/mL). All tests were performed in triplicate. The MIC value was estimated after 18 h of incubation at 37 °C in MHB using resazurin (Sigma-Aldrich, Darmstadt, Germany) [11]. To exclude an inhibitory effect of Tween 80 and DMSO on the MRSA strain growth, control assays with MHB and MHB containing 1% (*v*/*v*) Tween 80 or 2% (*v*/*v*) DMSO were conducted.

The subsequent step of the optimization assay was to analyze the interaction between EOCs and β-lactam antibiotics using the checkerboard method as previously described [11]. Briefly, each well contained the appropriate concentration of EOC (25 μL), β-lactam antibiotic (25 μL), and 50 μL of staphylococcal suspension (10^6^ CFU/mL). Then, 96-well plates were incubated for 18 h at 37 °C in aerobic conditions. All tests were performed in duplicate. For each replicate, fractional inhibitory concentration indices (FICI) were calculated using the following equations:

(1) $$\mathrm{FIC}\;=\;\frac{\mathrm{MIC}\;\mathrm{of}\;\mathrm{EOC}\;\mathrm{or}\;\beta \text{-}\mathrm{lactam}\;\mathrm{antibiotic}\;\mathrm{in}\;\mathrm{combination}\;}{\mathrm{MIC}\;\mathrm{of}\;\mathrm{EOC}\;\mathrm{or}\;\beta \text{-}\mathrm{lactam}\;\mathrm{antibiotic}\;\mathrm{alone}},$$

(2) FICI = FIC of EOC + FIC of β-lactam antibiotic

Results were interpreted as follows: synergism (FICI < 0.5), addition (0.5 ≤ FICI ≤ 1.0), indifference (1.1 < FICI ≤ 4.0), or antagonism (FICI > 4.0).

The final step of the optimization assay was based on determination and choosing the best (revealed synergistic and additive effects) combinations of EOC with β-lactam antibiotic. Obtained combinations were studied against all MRSA isolates.

### 4.7. Determination of MIC and FICI of Chemicals against Isolates

The MIC and FICI values of the EOCs and β-lactam antibiotics against isolates were determined according to the same method as that mentioned in Section 4.6. The MIC and FICI values of chemicals were estimated only for EOCs which showed the best combination (synergistic or additive effect) with β-lactam antibiotics received in the optimization assay using the *S. aureus* ATCC 43300 strain. All tests were performed in triplicate.

### 4.8. S. aureus Strains—FTIR Spectroscopic Measurements

FTIR spectroscopy analyses were conducted using staphylococcal colonies as described in our previous studies [11,13]. Briefly, after overnight culturing of strains at 37 °C, grown colonies were transferred directly to the Eppendorf tube, washed three times using PBS, and adjusted to 1.2 × 10^9^ CFU/mL. Each sample was dried and scanned at a range between 650 cm^−1^ and 4000 cm^−1^ (64 scans and 1 cm^−1^ resolution). The obtained spectra were normalized, baseline-corrected, and analyzed using SPECTRUM software (v10, Perkin Elmer, Waltham, MA, USA).

### 4.9. Statistical Analysis

All data were expressed as mean ± standard deviation (SD).

## 5. Conclusions

In conclusion, it could be said that the search for new substances with antibacterial properties is a result of the risks associated with antibiotic resistance. The current study confirms that changes in phosphodiester (especially with high-level β-lactam antibiotic-resistant strains), -OH, -CH_2_, and -CH_3_ groups may change the interactions with these antibiotics. Moreover, it was also concluded that linalyl acetate may be a potential compound with antibacterial activity used in combination with methicillin against both low- and high-level β-lactam antibiotic-resistant MRSA strains. This may be related to the presence of two methoxy (CH_3_O-) moieties in the methicillin molecule. Nevertheless, further studies are needed to explain the mechanism of the action of these two combined molecules. Direct therapy with linalyl acetate in combination with methicillin may become an alternative treatment method for staphylococcal infections caused by MRSA strains. However, this unconventional therapy must be preceded by numerous cytotoxicity tests, especially for the selection of an appropriate, safe concentration of the linalyl acetate-methicillin combination dose and time exposure studies. Our next step will be based on in vivo evaluation of the efficacy of the concentrations of the linalyl acetate-methicillin combination obtained in the present study.

## Figures and Tables

**Table 1:** Characteristics of *Staphylococcus aureus* strains.

Strain	Isolation Source	Susceptibility Testing					Phenotypic Resistance
		GE	CIP	FOX	E	CC	
*S. aureus* ATCC 43300	ATCC (clinical isolate)	R	S	R	R	R	MRSA, cMLS_B_
1	Surgical wound	R	R	R	R	R	MRSA, cMLS_B_
2	BAL	R	R	R	R	R	MRSA, cMLS_B_
3	BAL	R	R	R	R	R	MRSA, cMLS_B_
4	Surgical wound	R	R	R	R	R	MRSA, iMLS_B_
5	Surgical wound	R	R	R	R	R	MRSA, cMLS_B_
6	Urine	R	R	R	R	R	MRSA, cMLS_B_
7	Surgical wound	R	R	R	R	R	MRSA, cMLS_B_
8	Surgical wound	R	R	R	R	R	MRSA, cMLS_B_

ATCC—American Type Culture Collection, BAL—bronchoalveolar lavage, S—susceptible, R—resistant, GE—gentamicin, CIP—ciprofloxacin, FOX—cefoxitin, E—erythromycin, CC—clindamycin, MRSA—methicillin-resistant *S. aureus*, cMLS_B_—phenotypes expressing resistance to constitutive macrolides, lincosamides, and streptogramins B, iMLS_B_—phenotypes expressing resistance to inducible macrolides, lincosamides, and streptogramins B.

**Table 2:** Fractional inhibitory concentration (FIC) and FIC indices (FICIs) of pairs of essential oil compounds (EOCs) and β-lactam antibiotics (βLAs) against *Staphylococcus aureus* ATCC 43300 strain.

EOC–βLA	MICo	MICc	FIC	FICI	Type of Interaction
Methicillin–1,8-Cineole					
Methicillin (mg/L)	7.8 ± 0.0	7.8 ± 0.0	1.0	2.0	Indifference
1,8-Cineole (mg/mL)	115.1 ± 0.0	115.1 ± 0.0	1.0		
Methicillin–Eugenol					
Methicillin (mg/L)	7.8 ± 0.0	7.8 ± 0.0	1.0	2.5	Indifference
Eugenol (mg/mL)	11.1 ± 4.8	16.7 ± 0.0	1.5		
Methicillin–Carvacrol					
Methicillin (mg/L)	7.8 ± 0.0	7.8 ± 0.0	1.0	3.4	Indifference
Carvacrol (mg/mL)	3.2 ± 1.1	7.6 ± 0.0	2.4		
Methicillin–Linalool					
Methicillin (mg/L)	7.8 ± 0.0	7.8 ± 0.0	1.0	2.0	Indifference
Linalool (mg/mL)	6.8 ± 0.0	6.8 ± 0.0	1.0		
Methicillin–Linalyl acetate					
Methicillin (mg/L)	7.8 ± 0.0	2.0 ± 0.0	0.3	0.4	Synergy
Linalyl acetate (mg/mL)	46.9 ± 16.3	7.0 ± 0.0	0.1		
Methicillin–*trans*-Anethole					
Methicillin (mg/L)	7.8 ± 0.0	7.8 ± 0.0	1.0	2.0	Indifference
*trans*-Anethole (mg/mL)	494.0 ± 0.0	494.0 ± 0.0	1.0		
Methicillin–Thymol					
Methicillin (mg/L)	7.8 ± 0.0	15.6 ± 0.0	2.0	5.0	Antagonism
Thymol (mg/mL)	0.8 ± 0.4	2.5 ± 0.0	3.0		
Methicillin–Menthone					
Methicillin (mg/L)	7.8 ± 0.0	31.3 ± 0.0	4.0	6.0	Antagonism
Menthone (mg/mL)	27.9 ± 0.0	55.8 ± 0.0	2.0		
Penicillin G–1,8-Cineole					
Penicillin G (mg/L)	3.9 ± 0.0	0.2 ± 0.0	0.05	0.1	Synergy
1,8-Cineole (mg/mL)	115.1 ± 0.0	3.6 ± 0.0	0.03		
Penicillin G–Eugenol					
Penicillin G (mg/L)	3.9 ± 0.0	2.0 ± 0.0	0.5	1.2	Indifference
Eugenol (mg/mL)	11.1 ± 4.8	8.3 ± 0.0	0.7		
Penicillin G–Carvacrol					
Penicillin G (mg/L)	3.9 ± 0.0	2.0 ± 0.0	0.5	1.7	Indifference
Carvacrol (mg/mL)	3.2 ± 1.1	3.8 ± 0.0	1.2		
Penicillin G–Linalool					
Penicillin G (mg/L)	3.9 ± 0.0	2.0 ± 0.0	0.5	1.5	Indifference
Linalool (mg/mL)	6.8 ± 0.0	6.8 ± 0.0	1.0		
Penicillin G–Linalyl acetate					
Penicillin G (mg/L)	3.9 ± 0.0	2.0 ± 0.0	0.5	0.6	Addition
Linalyl acetate (mg/mL)	46.9 ± 16.3	3.5 ± 0.0	0.1		
Penicillin G–*trans*-Anethole					
Penicillin G (mg/L)	3.9 ± 0.0	3.9 ± 0.0	1.0	1.5	Indifference
*trans*-Anethole (mg/mL)	494.0 ± 0.0	247.0 ± 0.0	0.5		
Penicillin G–Thymol					
Penicillin G (mg/L)	3.9 ± 0.0	2.0 ± 0.0	0.5	1.3	Indifference
Thymol (mg/mL)	0.8 ± 0.4	0.6 ± 0.0	0.8		
Penicillin G–Menthone					
Penicillin G (mg/L)	3.9 ± 0.0	2.0 ± 0.0	0.5	1.5	Indifference
Menthone (mg/mL)	27.9 ± 0.0	27.9 ± 0.0	1.0		

MICo, minimal inhibitory concentration of EOC or βLA; MICc, minimal inhibitory concentration of EOC/βLA combination. FIC index = FIC of EOC + FIC of βLA. FICI < 0.5, synergy; 0.5 ≤ FICI ≤ 1.0, addition; 1.1 < FICI ≤ 4.0, indifference; FICI > 4.0, antagonism. Values are expressed as mean ± standard deviation.

**Table 3:** Fractional inhibitory concentration (FIC) and FIC indices (FICIs) of pairs of essential oil compounds (EOCs) and β-lactam antibiotics (βLAs) against MRSA strains.

Strain	EOC–βLA	MICo	MICc	FIC	FICI	Type of Interaction
1	Methicillin–Linalyl acetate					
	Methicillin (mg/L)	1000.0 ± 0.0	500.0 ± 0.0	0.5	1.0	Addition
	Linalyl acetate (mg/mL)	28.2 ± 0.0	14.1 ± 0.0	0.5		
	Penicillin G–Linalyl acetate					
	Penicillin G (mg/L)	125.0 ± 0.0	250.0 ± 0.0	2.0	4.0	Indifference
	Linalyl acetate (mg/mL)	28.2 ± 0.0	56.3 ± 0.0	2.0		
	Penicillin G–1,8-Cineole					
	Penicillin G (mg/L)	125.0 ± 0.0	250.0 ± 0.0	2.0	4.0	Indifference
	1,8-Cineole (mg/mL)	28.8 ± 0.0	57.6 ± 0.0	2.0		
2	Methicillin–Linalyl acetate					
	Methicillin (mg/L)	1000.0 ± 0.0	62.5 ± 0.0	0.1	0.2	Synergy
	Linalyl acetate (mg/mL)	112.6 ± 0.0	14.1 ± 0.0	0.1		
	Penicillin G–Linalyl acetate					
	Penicillin G (mg/L)	31.3 ± 0.0	93.9 ± 0.0	3.0	5.0	Antagonism
	Linalyl acetate (mg/mL)	112.6 ± 0.0	225.3 ± 0.0	2.0		
	Penicillin G–1,8-Cineole					
	Penicillin G (mg/L)	31.3 ± 0.0	93.9 ± 0.0	3.0	5.0	Antagonism
	1,8-Cineole (mg/mL)	28.8 ± 0.0	57.6 ± 4.2	2.0		
3	Methicillin–Linalyl acetate					
	Methicillin (mg/L)	1000.0 ± 0.0	125.0 ± 0.0	0.1	0.4	Synergy
	Linalyl acetate (mg/mL)	112.6 ± 0.0	28.2 ± 0.0	0.3		
	Penicillin G–Linalyl acetate					
	Penicillin G (mg/L)	31.3 ± 0.0	62.5 ± 0.0	2.0	3.0	Indifference
	Linalyl acetate (mg/mL)	112.6 ± 0.0	112.6 ± 0.0	1.0		
	Penicillin G–1,8-Cineole					
	Penicillin G (mg/L)	31.3 ± 0.0	62.5 ± 0.0	2.0	5.0	Antagonism
	1,8-Cineole (mg/mL)	28.8 ± 0.0	86.4 ± 0.0	3.0		
4	Methicillin–Linalyl acetate					
	Methicillin (mg/L)	31.3 ± 0.0	15.6 ± 0.0	0.5	1.0	Addition
	Linalyl acetate (mg/mL)	112.6 ± 0.0	56.3 ± 0.0	0.5		
	Penicillin G–Linalyl acetate					
	Penicillin G (mg/L)	3.9 ± 0.0	2.0 ± 0.0	0.5	0.8	Addition
	Linalyl acetate (mg/mL)	112.6 ± 0.0	28.2 ± 0.0	0.3		
	Penicillin G–1,8-Cineole					
	Penicillin G (mg/L)	3.9 ± 0.0	0.5 ± 0.0	0.1	0.2	Synergy
	1,8-Cineole (mg/mL)	57.6 ± 0.0	7.2 ± 0.0	0.1		
5	Methicillin–Linalyl acetate					
	Methicillin (mg/L)	1000.0 ± 0.0	500.0 ± 0.0	0.5	1.0	Addition
	Linalyl acetate (mg/mL)	56.3 ± 0.0	28.2 ± 0.0	0.5		
	Penicillin G–Linalyl acetate					
	Penicillin G (mg/L)	125.0 ± 0.0	250.0 ± 0.0	2.0	5.0	Antagonism
	Linalyl acetate (mg/mL)	56.3 ± 0.0	168.9 ± 10.0	3.0		
	Penicillin G–1,8-Cineole					
	Penicillin G (mg/L)	125.0 ± 0.0	250.0 ± 0.0	2.0	4.0	Indifference
	1,8-Cineole (mg/mL)	57.6 ± 0.0	115.2 ± 0.0	2.0		
6	Methicillin–Linalyl acetate					
	Methicillin (mg/L)	7.8 ± 0.0	3.9 ± 0.0	0.5	0.8	Addition
	Linalyl acetate (mg/mL)	56.3 ± 0.0	14.1 ± 0.0	0.3		
	Penicillin G–Linalyl acetate					
	Penicillin G (mg/L)	3.9 ± 0.0	1.0 ± 0.0	0.3	0.6	Addition
	Linalyl acetate (mg/mL)	56.3 ± 0.0	14.1 ± 0.0	0.3		
	Penicillin G–1,8-Cineole					
	Penicillin G (mg/L)	3.9 ± 0.0	0.5 ± 0.0	0.1	0.2	Synergy
	1,8-Cineole (mg/mL)	57.6 ± 0.0	7.2 ± 0.0	0.1		
7	Methicillin–Linalyl acetate					
	Methicillin (mg/L)	1000.0 ± 0.0	250.0 ± 0.0	0.3	0.6	Addition
	Linalyl acetate (mg/mL)	56.3 ± 0.0	14.1 ± 0.0	0.3		
	Penicillin G–Linalyl acetate					
	Penicillin G (mg/L)	31.3 ± 0.0	31.3 ± 0.0	1.0	2.0	Indifference
	Linalyl acetate (mg/mL)	56.3 ± 0.0	56.3 ± 0.0	1.0		
	Penicillin G–1,8-Cineole					
	Penicillin G (mg/L)	31.3 ± 0.0	31.3 ± 0.0	1.0	3.0	Indifference
	1,8-Cineole (mg/mL)	28.8 ± 0.0	57.6 ± 0.0	2.0		
8	Methicillin–Linalyl acetate					
	Methicillin (mg/L)	62.5 ± 0.0	15.6 ± 0.0	0.3	0.6	Addition
	Linalyl acetate (mg/mL)	112.6 ± 0.0	28.2 ± 0.0	0.3		
	Penicillin G–Linalyl acetate					
	Penicillin G (mg/L)	3.9 ± 0.0	0.5 ± 0.0	0.1	0.6	Addition
	Linalyl acetate (mg/mL)	112.6 ± 0.0	56.3 ± 0.0	0.5		
	Penicillin G–1,8-Cineole					
	Penicillin G (mg/L)	3.9 ± 0.0	0.2 ± 0.0	0.1	0.4	Synergy
	1,8-Cineole (mg/mL)	57.6 ± 0.0	14.4 ± 0.0	0.3		

MICo, minimal inhibitory concentration of EOC or βLA; MICc, minimal inhibitory concentration of EOC/βLA combination. FIC index = FIC of EOC + FIC of βLA. FICI < 0.5, synergy; 0.5 ≤ FICI ≤ 1.0, addition; 1.1 < FICI ≤ 4.0, indifference; FICI > 4.0, antagonism. Values are expressed as mean ± standard deviation.

## Abbreviations

EOs	essential oils
EOCs	essential oil compounds
FICI	fractional inhibitory concentration index
FTIR	Fourier-transform infrared spectroscopy
MIC	minimal inhibitory concentration
MLS_B_	phenotype expressing resistance to macrolides, lincosamides, and streptogramins B
MRSA	methicillin-resistant *Staphylococcus aureus*
PCR	polymerase chain reaction
PFGE	pulsed-field gel electrophoresis
